# Supplementary material for: Neural evidence for lexical parafoveal processing
Source: Nat Commun. 2021 Sep 2;12:5234. doi: 10.1038/s41467-021-25571-x (PMC8413448; doi:10.1038/s41467-021-25571-x)
Supplement: Supplementary file 1 — Supplementary Information [file 41467_2021_25571_MOESM1_ESM.pdf]

Supplementary Information

Neural evidence for lexical parafoveal processing

Yali Pan[1,2*], Steven Frisson[1,2] and Ole Jensen[1,2]

[1] Centre for Human Brain Health, University of Birmingham, Birmingham B15 2TT, United Kingdom

[2] School of Psychology, University of Birmingham, Birmingham B15 2TT, United Kingdom

***Correspondence:** yalipan666@gmail.com

List of Contents

Supplementary table 1
Supplementary Figures 1-7
Supplementary notes
Supplementary methods
Supplementary references

Supplementary table 1. Orthographic variables for the target words.

	Low lexical freq target	High lexical freq target	*t* values
Bigram type freq	54.0 (41.2)	61.1 (46.2)	-1.741 (p = 0.083)
Bigram token freq	979.2 (737.2)	1332.7 (886.7)	-4.816 (p = 2.6e-06)
Trigram type freq	9.4 (12.2)	11.6 (14.0)	-1.810 (p = 0.072)
Trigram token freq	153.7 (288.0)	320.6 (334.2)	-6.060 (p = 5.2e-09)
Neighborhood size	1.9 (2.4)	2.4 (2.6)	-2.507 (p = 0.013)

Note. All measures are mean values estimated from the N-watch program[1]. The standard deviations are in parentheses. Pair-wise t-tests were conducted for each measure between low and high lexical frequency target words, the t-values are shown in the third column with the p-values in parentheses. Source data are provided as a Source Data file.

Bigrams refer to two successive letters in a string, e.g. bigrams for the word *edge* are *ed, dg,* and *ge*. For each bigram, the type frequency is the number of all 4-letter words that contain this bigram in the same position – length and position sensitive; e.g., the type frequency for *ed* is 4 (*eddy, edge, edgy,* and *edit*). The token frequency for each bigram is the sum of the word frequencies for all types; e.g., the token frequency for *ed* is 81, the sum of word frequencies for these four words (*eddy, edge, edgy,* and *edit*). The bigram type/token frequency for the whole word is the averaged type/token frequencies over all bigrams. Trigram is three successive letters in a string, e.g., trigrams for the word *edge* are *edg* and *dge*. The trigram type and token frequency are calculated in a similar way as for bigrams.

Neighbourhood size of a string indicates how many words can be formed by just substituting one letter in the string.

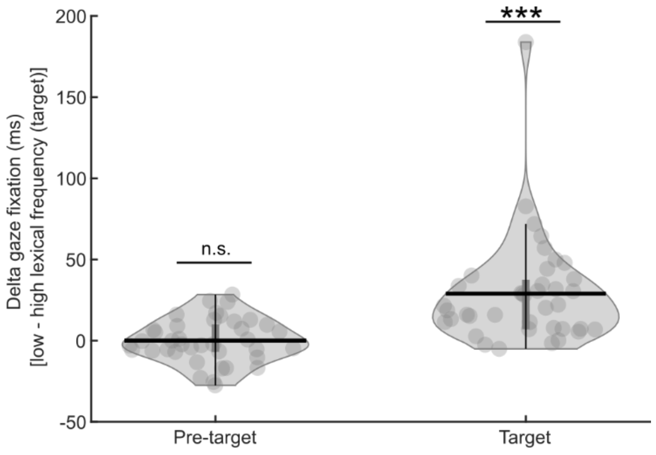

Supplementary Figure 1 | Gaze-duration difference for pre-target and target words. Gaze duration refers to the sum of all first-pass fixations for a given word. The differences for low minus high lexical frequency of the target words were calculated. A significant lexical frequency effect was only observed for target words, with longer gaze durations for low- compared with high-lexical frequency (*** $p = 2.88\times10e{-6}$, $n = 39$, two-tailed paired t-test). We observed no significant gaze duration difference for pre-target words ($p = 0.977$, $n = 39$, two-tailed paired t-test). The horizontal bar in the violin plot indicates mean value; each dot represents one participant. This finding supports the conclusion of Fig. 2. Source data are provided as a Source Data file.

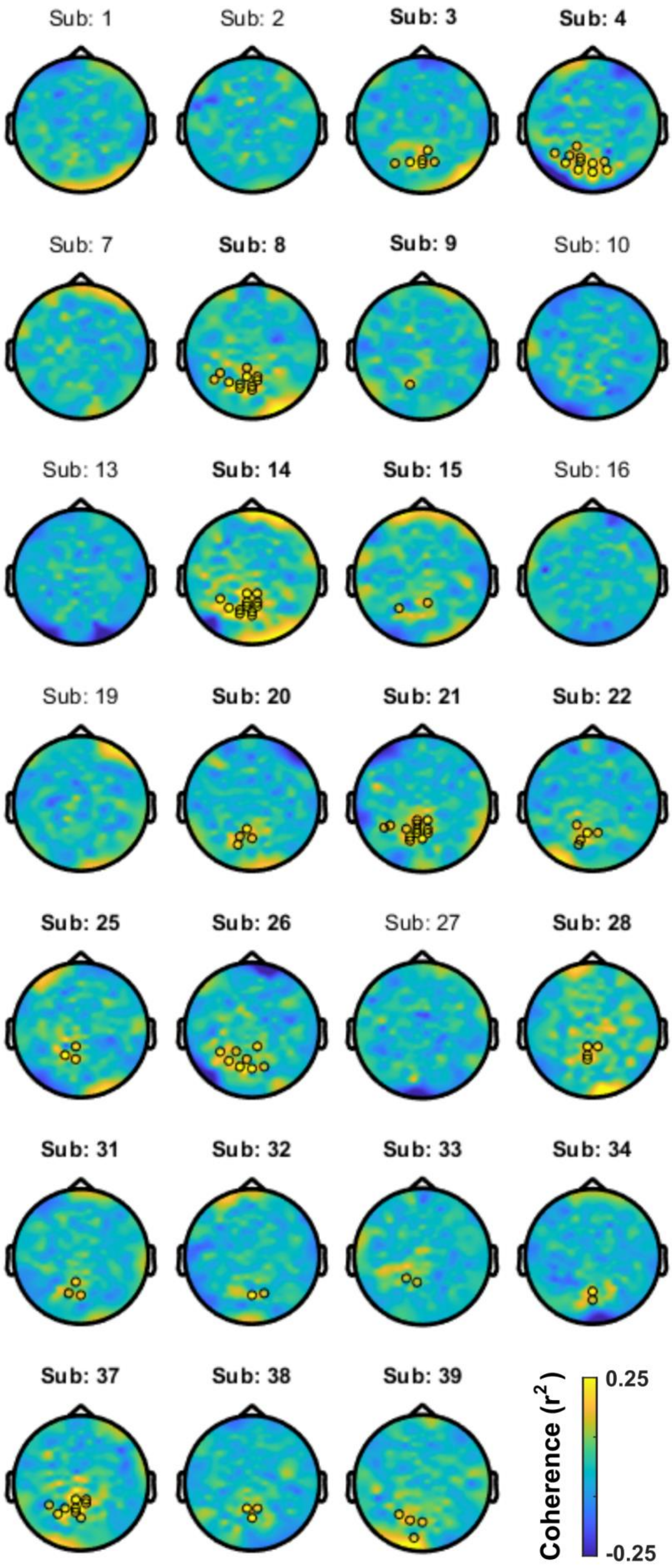
Sub: 1
Sub: 2
Sub: 3
Sub: 4
Sub: 5
Sub: 6
Sub: 7
Sub: 8
Sub: 9
Sub: 10
Sub: 11
Sub: 12
Sub: 13
Sub: 14
Sub: 15
Sub: 16
Sub: 17
Sub: 18
Sub: 19
Sub: 20
Sub: 21
Sub: 22
Sub: 23
Sub: 24
Sub: 25
Sub: 26
Sub: 27
Sub: 28
Sub: 29
Sub: 30
Sub: 31
Sub: 32
Sub: 33
Sub: 34
Sub: 35
Sub: 36
Sub: 37
Sub: 38
Sub: 39
Coherence (r^2)
0.25
-0.25

Supplementary Figure 2 | Topography of significant tagging response sensors for each participant. A Monte-Carlo based permutation test was performed to select sensors that showed stronger coherence to the tagging signal during the pre-target fixation period compared with the baseline period (no flicker). Significant sensors are marked with circles in the topography, with the values indicating coherence difference (pre-target minus baseline). Participants who had significant tagging response sensors are shown with a bold subtitle. Further coherence analyses (Fig.4, Fig.5, and Supplementary Figure 4) were based on the marked sensors.

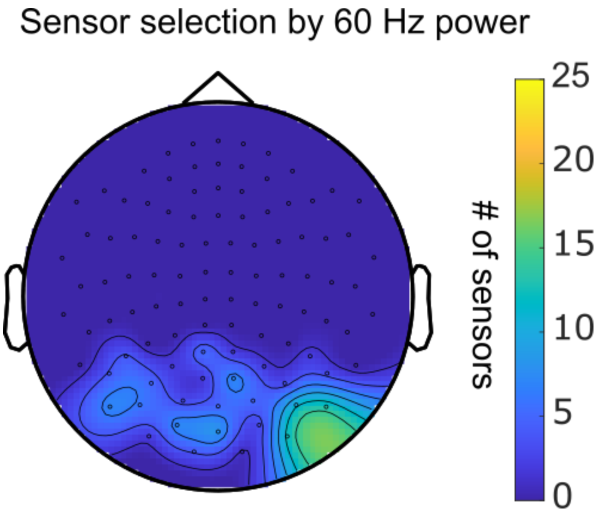

Supplementary Figure 3 | Topography for tagging response sensors selected by 60Hz power. Topography for sensors from all participants that showed stronger 60 Hz power during the pre-target period (flicker) compared with the baseline period (no-flicker, n=26). It was the same sensor selection procedure as in Figure 3, but used the 60 Hz power instead.

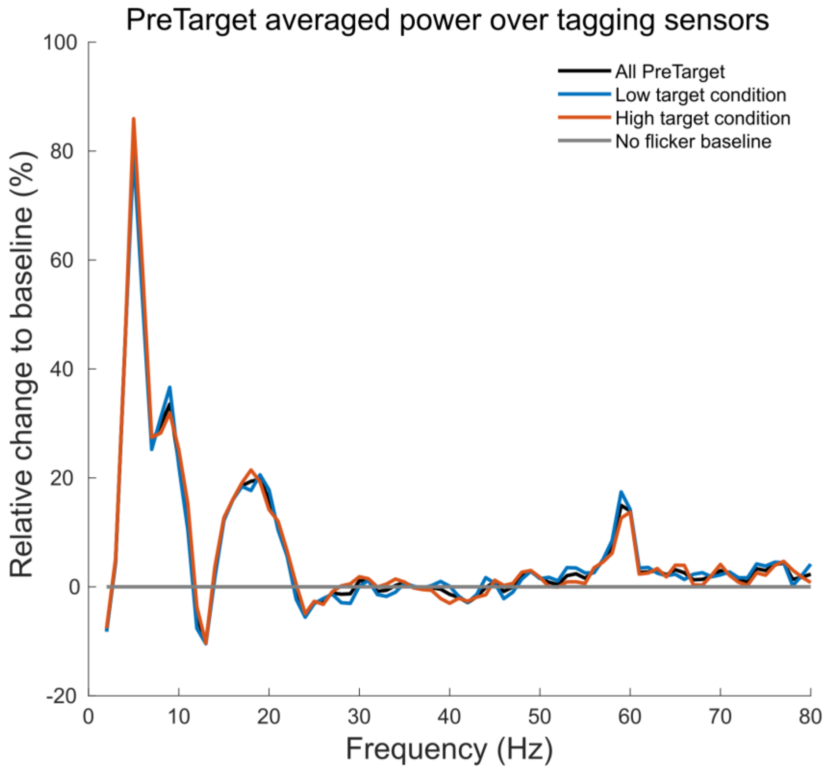

Supplementary Figure 4 | Power spectrum for the pre-target fixation period over significant tagging response sensors. For each participant (n = 26), power was calculated from all tagging response sensors (from 2 to 80 Hz; 1 Hz steps; Hanning taper). This was conducted for all pre-target epochs (indicated by the black line), pre-target words that were followed by low lexical frequency targets (blue line), pre-target words that were followed by high lexical frequency targets (orange line), and the baseline epochs (grey line). Next, power values in each condition were averaged across all tagging response sensors and then averaged across all participants. These power values were then transformed as the relative change with baseline power to get rid of the 1/f component (at each frequency point, (Pow – $Pow_{baseline}$)/$Pow_{baseline}$). We can see a clear peak around the tagging frequency at 60 Hz for both pre-target word conditions but not the baseline condition, indicating a stable tagging response to the flickering target words in the parafovea.

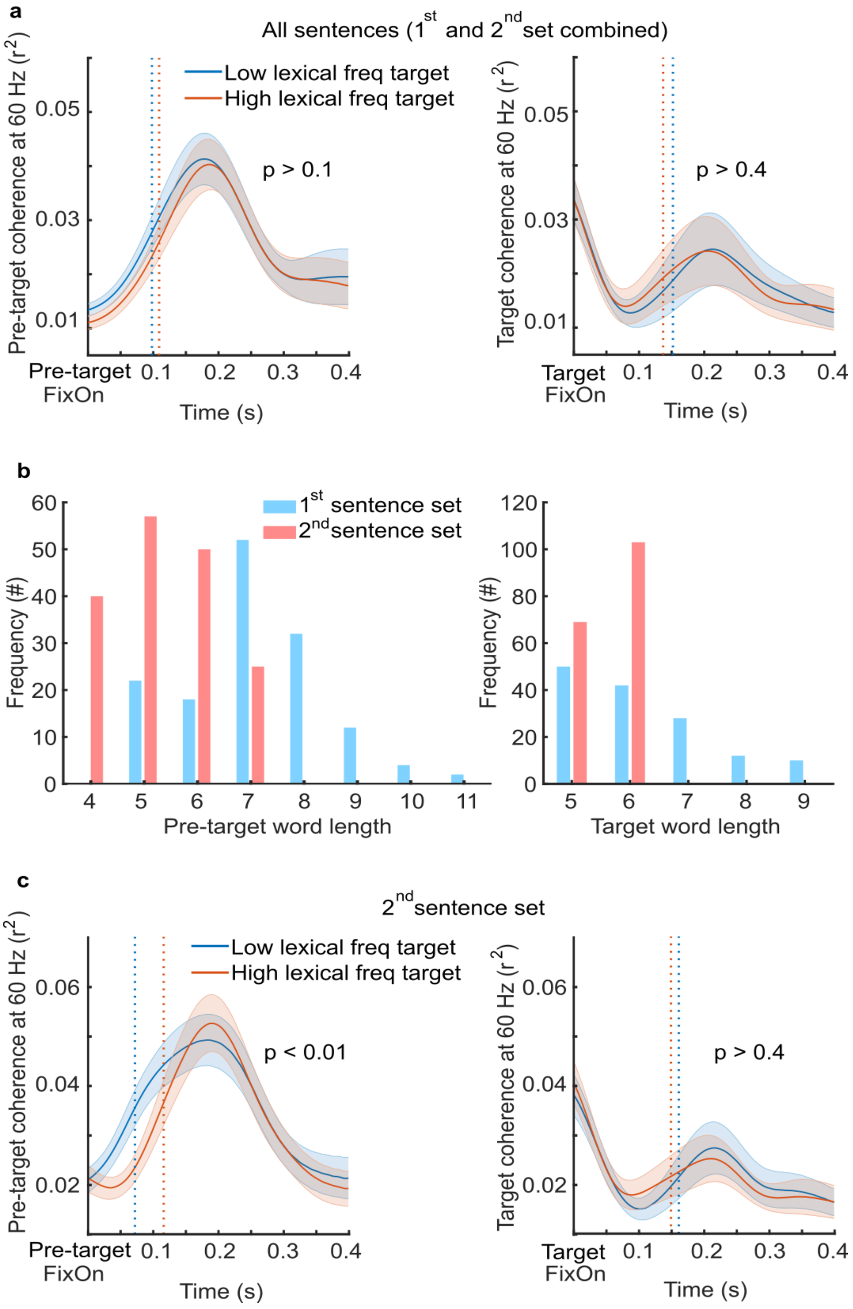

Supplementary Figure 5 | Pre-target coherence onset latency was modulated by parafoveal target lexical frequency in the short-word sentence set (2nd Set). A Jackknife-based method was used to identify the onset latency for 60 Hz coherence at the group level. Here onset latency refers to the time when coherence reached half maximum, denoted by the dotted lines. (**a**) Each participant read two sets of sentences; for the combined sentence set, no significant difference was found in either pre-target fixation (left panel, $p > 0.1$, $n = 26$, two-tailed paired t-test) or target fixation (right panel, $p > 0.4$, $n = 26$, two-tailed paired t-test). For panel **a** and **c**, the shaded areas represent standard error over 26 participants. (**b**) The histogram of word length showed that in the 2nd sentence set (red bars), both pre-target and target words were shorter compared with the 1st sentence set (blue bars). (**c**) For the 2nd sentence set, a significant coherence onset difference was found in pre-target fixation interval (left panel, $p < 0.01$, $n = 26$, two-tailed paired t-test), but not in target fixation interval (right panel, $p > 0.4$, $n = 26$, two-tailed paired t-test).

Supplementary notes

Lexical parafoveal previewing evidence from coherence onset latency

Did the lexical frequency of the target word affect onset latency for parafoveal previewing? The pre-target coherence onset latency was defined by the time to reach its half maximum. We then used a Jackknife procedure[2] to statistically evaluate if the coherence onset was significantly different for low compared with high lexical frequency target words. While no statistical difference was found for the combined sets of sentences (Supplementary Figure 5a left panel, $t_{(25)} = -1.69$, $p > 0.1$, two-tailed pairwise t-test), we did find a significant difference for the set with shorter pre-target and target words (2nd sentence set; see Supplementary Figure 5c left panel, $t_{(25)} = -2.85$, $p < 0.01$, two-tailed pairwise t-test). Importantly, this effect was already visible around the first 100ms of the pre-target fixation (72ms for low lexical frequency target; 116ms for high lexical frequency target). The null finding for the combined sentence set might be explained by the longer and more varying length of the pre-target and target words (Supplementary Figure 5b). The target words in the 2nd sentence set might be too long to be previewed within the effective perceptual span, which is around 12 letters in reading direction[3–5]. The faster onset for the low lexical frequency target indicates that parafoveal neuronal processing is modulated in time by upcoming lexical information as well, but this effect is only visible when pre-target and target words are relatively short. In particular we find that the neuronal responses for the low-frequency words tend to occur earlier than for high-frequency words. This might seem at odds with the expectation that lexical processing is faster for high than low-frequency words. However, the exact onset of the neuronal response should be interpreted with caution, as it is co-modulated by the magnitude of the response.

Jackknife-based method for onset latency

We used a leave-one-out Jackknife-based method[2] to assess the onset latency difference for the pre-target and target coherence separately with respect to the target lexical frequency. During each iteration, one randomly chosen participant was left out, and the averaged coherences for low and high target lexical frequency condition were calculated over the remaining participants. Then, the coherence onset latencies were computed for both conditions. Here, the onset latency was defined as the time point when the averaged coherence value reached its half-maximum ($\mathrm{coh_{min}} + (\mathrm{coh_{max}} - \mathrm{coh_{min}})/2$). We computed the onset latency difference by subtracting the onset latency for the low target lexical frequency condition from the high condition. After twenty-six iteration (n = 26), onset latency differences from all these subsamples were pooled together to estimate a standard error (S_D) using the following equation:

$$S_D = \sqrt{\frac{n-1}{n} \cdot \sum_{i=1}^{n} \left(D_{-i} - \bar{J}\right)^2}$$

where the $\bar{J}$ is the averaged onset latency difference over all the subsamples, D_{-i} is the coherence difference obtained from the subsample when participant i was left out, n is the number of participants. We also computed the onset latency difference from the overall sample set (without leaving any participant out), and divided it by the S_D to obtain its *t*-value. A standard t table provided the statistical significance for the coherence onset latency difference between high and low target lexical frequency conditions. This procedure was done for both pre-target and target segments to conduct a statistical test for the coherence onset latency difference between both target lexical frequency conditions as shown in Supplementary Figures 5a and 5c. (for details about the Jackknife-based method for measuring onset latency differences, please see Miller et al., 1998[2]).

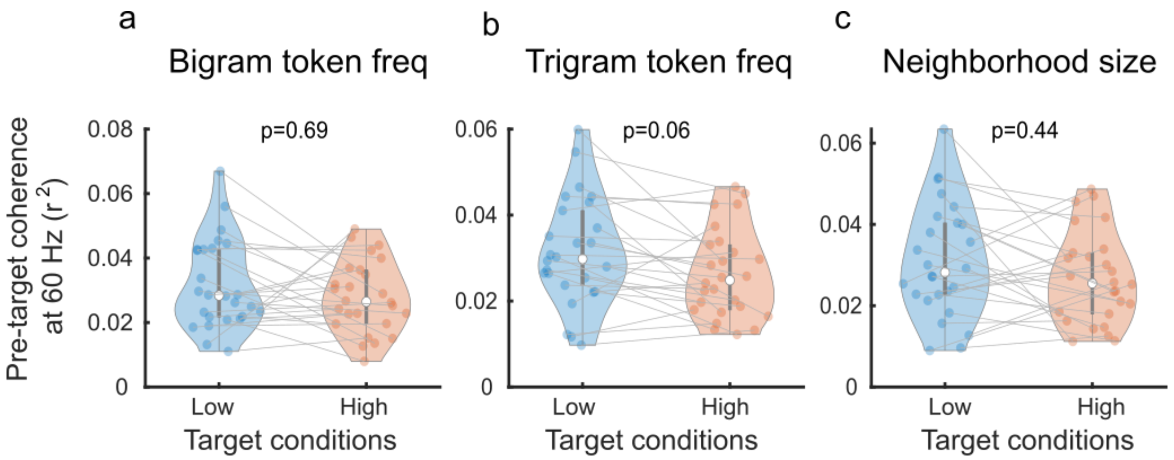

Supplementary Figure 6 | Three control analyses of orthographic parafoveal previewing effect. Target words were seprated by their (**a**) bigram token frequency, (**b**) trigram token freuency, and (**c**) neighborhood size (for definations of these variables please see Supplementary table). For each target condition, pre-target coherence at 60 Hz was estimated from the same tagging response sensors with the same method as used in Figure 4b. Three ttest were conducted for all three orthographic variables to search for any possible significance for pre-target coherecen, but no significance was found (p values were Bonferroni corrected, n = 26, two-sided pairwise t-test). Eact dot presents one participant. Freq, frequency. Source data are provided as a Source Data file.

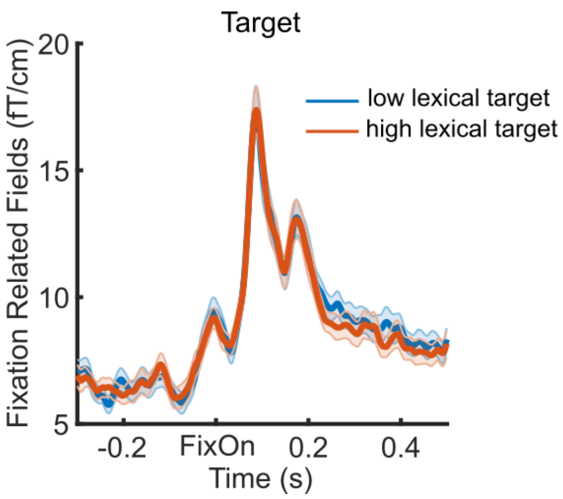

Supplementary Figure 7. Fixation-related fields (FRFs) for target words. We performed the same FRFs analysis for target fixations as in Fig. 6, but found no significant difference with respect to lexical frequency (n = 39, cluster-based permutation with $p < 0.05$, two-tailed pairwise t-test, 1000 permutations). Here we show the averaged FRFs for low (solid blue line) and high (solid orange line) lexical target words over the sensors shown in Fig. 6a. The shaded areas represent standard error over 39 participants.

Supplementary methods

Two Sentence sets

For a full list of sentences that used in the experiment, please see Experimental sentence set #1 and #2.

Set #1

We made 142 sentences with 71 target pairs (low/high word frequency) embedded. For each sentence, the pre-target, target and post-target word were in the same structure as adjective + noun + verb. For each target pair, two different sentence frames were made and participant read both target words. For example, for target pair **waltz/music** (low/high frequency), one participant read version A, another one read version B (see below, targets were bold for illustration, but not bold in experiment).

A. Mike thought this difficult **waltz** received lots of criticism.
   It was obvious that the beautiful **music** captured her attention.
B. Mike thought this difficult **music** received lots of criticism.
   It was obvious that the beautiful **waltz** captured her attention.

Sentence sequence in version B was scramble from version A by shift the first and second half sentences. For both version, no more than 3 successive sentences were from the same target frequency condition.

Set #2

This sentence set was from Degno et al., 2019[2], after removing the sentences that contained the same pre-target or target words as in sentence set #1, 86 sentences left. In each sentence, two target words both in low or high frequency were embedded (see below, version A contained low frequency targets, version B contained high frequency targets).

A. I felt quite **bleak** after discussing that really **risky** subject with Paul.
B. I felt quite **weird** after discussing that really **nasty** subject with Paul.

Participant read either version A or B. Same control for sentence sequence as in set #1.

Pre-tests for target predictability and sentence plausibility

We carried out behavioural pre-tests for the 1st sentence set only; for pre-test results for the 2nd set please see Degno et al., 2019[6]. The participants in the pre-tests did not participate in the MEG session.

Predictability

A cloze test was performed using the sentence fragment up to but not including the target word. Participants were asked to read the sentence silently and then write down the first word/s that came to mind to complete the sentence. Example:

`Mike thought this difficult _________________`

If a target word (e.g. *waltz* or *music*) was generated by less than 10% of the participants, then the target was considered unpredictable. Twenty-two participants (1 male) took part in this pre-test, and 6 target words turned out to be highly predictable. These 6 target words were replaced and the predictability test was conducted again with another 22 participants (3 males). None of the target words were judged predictable.

Plausibility

Participants were instructed to rate how plausible (or acceptable) each sentence was. Plausibility was rated on a 7 point scale (see examples below). Sentences in the experiment were supposed to be highly plausible, in order to occupy the full range of the scale, we constructed 142 filler sentences with low plausibility, half of which were of middle plausibility (e.g., sentence 1 below) and another half were implausibility (e.g., sentence 3 below). In this example, sentence 2 was used in the experiment.

	Implausible Plausible<br>1 2 3 4 5 6 7
1. Kate said that she saw lots of stars twinkling in the sky at noon.	1 2 3 4 5 6 7
2. It was obvious that the beautiful music captured her attention.	1 2 3 4 5 6 7
3. Sarah used an adhesive to glue the traffic and just made it to the wedding.	1 2 3 4 5 6 7
……	1 2 3 4 5 6 7

For version A (n = 22, 2 males, 2 invalid for incomplete or careless responses), the plausibility rating for the experimental sentences 5.7 ± 0.5 (mean ± SD), which was

significantly higher than the filler sentences that were of low plausibility (two-tailed pair-wised $t_{(19)} = 23.98$, $p < 0.001$). For version B (n = 23, 4 males), the rating was 5.5 ± 0.8 (mean ± SD), which was also significantly higher than the filler sentences (two-tailed pair-wised $t_{(22)} = 16.68$, $p < 0.001$). These results showed that all the sentences in both versions were highly plausible.

Experimental sentence set #1

In total, 142 sentences embedded with 142 pairs of target (e.g., **music/waltz**). For illustration, high lexical frequency targets are shown in bold red font, while low lexical frequency targets are shown in bold black font. All targets were in normal black font in the experiment.

Every participant read all target pairs in different sentence frames. For example, participant A read "It was obvious that the beautiful **music** captured her attention." and "Mike thought this difficult **waltz** received lots of criticism."; while participant B read "It was obvious that the beautiful **waltz** captured her attention." and "Mike thought this difficult **music** received lots of criticism."

1. It was obvious that the beautiful **music/waltz** captured her attention.
2. Mike thought this difficult **waltz/music** received lots of criticism.
3. Before sleeping, the exhausted **student/steward** smoked a cigarette.
4. Last Wednesday, the energetic **steward/student** missed the flight.
5. The young man's shiny **vehicle/gondola** vanished slowly out of sight.
6. No-one knew why the shaky **gondola/vehicle** squeaked loudly at night.
7. People said that the ancient **church/mosque** remained nearly empty.
8. I heard that the largest **mosque/church** happened to be ruined.
9. In the animation, the noisy **chicken/rooster** chased the sparrows.
10. The old couple's plump **rooster/chicken** caught a lot of fat worms.
11. To the north, the clean **beach/dunes** stretched for many miles.
12. For the locals, the dirty **dunes/beach** triggered a political issue.

13. John said that the young **director/examiner** annoyed his friends.
14. Not surprisingly, the proud **examiner/director** refused the job.
15. In that film, the youthful **officer/refugee** entered the room calmly.
16. Just now, the careless **refugee/officer** ignored the sign and walked in.
17. Thankfully, the concerned **teacher/curator** pacified the boy quickly.
18. Finally, the attentive **curator/teacher** reported the incident to her boss.
19. The girl found the turkey's **wings/claws** bruised from the fighting.
20. The abscess on the parrot's **claws/wings** swelled to twice its size.
21. Last Sunday, a sizeable **group/squad** gathered in the city streets.
22. Apparently, a reckless **squad/group** startled some innocent bystanders.
23. In the dark of the night, a brown **horse/eagle** emerged from nowhere.
24. The government said that a black **eagle/horse** escaped from the zoo.
25. It turned out that the amazing **journey/banquet** lasted for six hours.
26. Ellen said that the outdoor **banquet/journey** turned out to be boring.
27. In the last few years, the northern **forest/meadow** decreased dramatically.
28. It revealed that the isolated **meadow/forest** flourished during the spring.
29. They had no idea that the transparent **liquid/lotion** shrinks all clothes.
30. She proved that the biochemical **lotion/liquid** spreads quickly in water.
31. Harriet saw the leather **shoes/belts** dumped in the fancy store.
32. Finally, she found the missing **belts/shoes/trees/ferns** infested with insects.
33. Yesterday, Linda found the strange **trees/ferns** infested with insects.
34. It was a good sign that the massive **ferns/trees** survived the cold winter.
35. After the meeting, the anxious **worker/tailor** sighed in the hallway.
36. Geoffrey said that the capable **tailor/worker** closed the door and left.
37. Sue said that the natural **water/tonic** relieved her headache quickly.
38. Lee said that the harmful **tonic/water** poisoned huge numbers of bugs.
39. In the big house, an enormous **painting/snapshot** adorned the bedroom.
40. He recalled that the delicate **snapshot/painting** brought tears to his eyes.
41. In this museum, the irregular **stones/jewels** glowed softly at night.
42. It's true that the exquisite **jewels/stones** traded well last year.

43. Straightaway, a drop of awful **coffee/iodine** stained his shirt.
44. In no time, a bit of nasty **iodine/coffee** spilled out of the bottle.
45. The barrister said the broken **knife/razor** confirmed the guilt of Jim.
46. Unfortunately, the silver **razor/knife** scratched the young man's palm.
47. In the first scene, the fearless **peasant/shooter** terrified the male lion.
48. Almost a decade ago, an ignorant **shooter/peasant** destroyed the log cabin.
49. Without anyone noticing, the starving **enemy/rover** nicked the cereal.
50. On a cold rainy night, the horrible **rover/enemy** roamed the cornfield.
51. It was big news that the prized **sheep/panda** produced a litter yesterday.
52. The zookeeper said that the lonely **panda/sheep** depended on him completely.
53. At yesterday's party, the vivid **flower/orchid** trembled in the wind.
54. Customers complained that the fresh **orchid/flower** withered very quickly.
55. Laura was told that the intense **storm/sleet** delayed the flight.
56. We were warned that the serious **sleet/storm** reduced visibility.
57. On Sunday evening, the hungry **clerk/nanny** cooked a beef patty.
58. Before dinner, the modest **nanny/clerk** tasted the tomato soup.
59. Due to the moist weather, the plain **bread/pasta** became mouldy quickly.
60. After a day of hard labour, the cheap **pasta/bread** looked very delicious.
61. Eyewitnesses said the angry **priest/cowboy** frightened the little girl.
62. Unexpectedly, the drunk **cowboy/priest** forewarned the neighbours.
63. Eventually, the German **servant/cobbler** disclosed all the details.
64. There was no way that the stupid **cobbler/servant** convinced anyone.
65. It was said that the honest **judge/miner** convened the committee meeting.
66. Last Christmas, the cheery **miner/judge** glimpsed the spectacular event.
67. Before the war, the renowned **general/rancher** assembled a large crowd.
68. In this film, the youthful **rancher/general** inherited a huge mansion.
69. On snowy days, the careful **driver/server** reminded himself to go slowly.
70. A minute later, the foolish **server/driver** provoked his big boss.
71. At home, the fatigued **manager/dentist** complained about being overworked.
72. Abruptly, the agitated **dentist/manager** terminated the conversation.

73. As usual, the emotional **captain/trainee** added excess sugar to his tea.
74. Unfortunately, the unskilled **trainee/captain** froze during the emergency.
75. In the lab, the composed **doctor/intern** examined the victim's body.
76. In the end, the seasoned **intern/doctor** selected the sharp scalpel.
77. Lily claimed that the shrewd **farmer/barber** avoided the nasty salesman.
78. As often happened, the joyous **barber/farmer** praised his charming wife.
79. Afterwards, the deliberate **artist/dancer** doubted the explanation.
80. Tom said the successful **dancer/artist** enjoyed our work very much.
81. The editor knew that the alert **president/statesman** revised the report.
82. In that meeting, the tough **statesman/president** imposed the new policy.
83. That night, the hateful **letter/memoir** distressed the pianist.
84. We heard that the curious **memoir/letter** implicated the drummer.
85. People knew that the haughty **principal/performer** despised his colleagues.
86. Around the corner, the liberal **performer/principal** rewarded the homeless.
87. No surprise, the worrying **writer/banker** neglected his vocation again.
88. On the TV, the innocent **banker/writer** justified himself to the public.
89. One day later, the cunning **agent/buyer** released the amended price.
90. Due to the crisis, the obscure **buyer/agent** suffered many hardships.
91. The panel doubted that the vicious **criminal/retailer** confessed the truth.
92. There is a rumour that the foolish **retailer/criminal** defrauded his lawyer.
93. Nobody knew when the excited **child/puppy** urinated on the floor.
94. Yesterday evening, the healthy **puppy/child** sprained its ankle.
95. Last weekend, the amiable **professor/sportsman** shared his experience.
96. In the end, the foreign **sportsman/professor** talked about his scandal.
97. The newspaper stated the unforeseen **accident/epidemic** caused fifty deaths.
98. Undeniably, the inevitable **epidemic/accident** showed the authorities a lot.
99. Last month, the beloved **minister/musician** denounced the mass murder.
100. This morning, the radical **musician/minister** advocated the new law.

101.He thought that the standard **engine/guitar** sounded very weird.
102.Bill noticed that the outmoded **guitar/engine** emitted bizarre noises.
103.In the open field, the dreaded **chief/ruler** executed the prisoner.
104.Last December, the sincere **ruler/chief** promised to end the conflict.
105.The old couple said that the humorous **guide/clown** brought lots of joy.
106.For two years, the tolerant **clown/guide** endured insults from his boss.
107.Reports said the crooked **police/robber** abused a person for several hours.
108.Last night, the nervous **robber/police** scared the lonely wanderer.
109.Early this century, the exotic **grass/maple** prevailed over the land.
110.In the cold north, the strong **maple/grass** prevented soil erosion greatly.
111.Scientists didn’t know why the lethal **cancer/plague** killed only men.
112.Results indicated that the deadly **plague/cancer** varied greatly widely.
113.Lily said that the unusual **island/canyon** belonged to her motherland.
114.Actually, the distant **canyon/island** provided a habitat for many animals.
115.Sam thought that the pathetic **story/video** portrayed the event truly.
116.Ben said that the romantic **video/story** described a miserable encounter.
117.The firemen found that the classic **train/sedan** obstructed the traffic.
118.It was good that the ancient **sedan/train** functioned well in the snow.
119.The noises coming from the nearby **coast/wharf** persisted the whole night.
120.Bright flashes from the remote **wharf/coast** navigated the sailors at sea.
121.Because of anxiety, the awkward **model/boxer** drank a lot of wine.
122.Before the contest, the unlucky **boxer/model** broke his right arm.
123.They agreed that the energetic **member/singer** planned the event well.
124.Karl thought that the deceitful **singer/member** donated nothing at all.
125.Jay felt that the popular **picture/cartoon** expressed a tinge of sadness.
126.It was said that the obscure **cartoon/picture** indicated something odd.
127.One lab found that the sweet **oranges/lychees** contained lots of vitamin E.

128. It is said that the local **lychees/oranges** dominated the fruit market.
129. Jack said that the premium **display/scanner** presented perfect halftones.
130. The ad said that the digital **scanner/display** sharpened images greatly.
131. Last year, this kind of electric **device/cooker** retailed for £50 each.
132. Jerry said that this ordinary **cooker/device** improved his quality of life
133. Studies concluded that vigorous **movement/canoeing** damaged our back muscles.
134. Bob said that moderate **canoeing/movement** reduced his anxiety effectively.
135. On holidays, the thrifty **secretary/librarian** stewed beans for dinner.
136. Last semester, the popular **librarian/secretary** taught a practical course.
137. This kind of complex **equipment/appliance** recorded room temperature precisely.
138. Eve said that this medical **appliance/equipment** required uninterrupted power.
139. Visible to the naked eye, the flaming **plane/comet** brightened the dark sky.
140. Within minutes, the falling **comet/plane** approached its maximum speed.
141. Jessie said the powerful **disease/malaria** continued to affect many children.
142. It was bad that the dreadful **malaria/disease** unsettled the best scientists.

Experimental sentence set #2

This sentence set is adapted from Degno et al., 2019[6]. After removing sentences that contain the same pre-target or target words, this set remains 86 sentences. Each sentence frame is embedded with two target words, both are of either high lexical frequency (red bold font) or

low lexical frequency (bold black font). All targets were in normal black font in the experiment.

For each sentence frame, participant A read it embedded with two high lexical frequency target words, while participant B read it embedded with two low lexical frequency target words. So, the two target words in a sentence are always of the same lexical condition.

1. I felt quite **weird/bleak** after discussing that really **nasty/risky** subject with Paul.
2. The purchase of a blue **heavy/plush** jumper led to another **random/futile** argument yesterday.
3. There were more **larger/quaint** buildings which were **mainly/ vastly** populated by students.
4. Susy bought some **steel/resin** bead bracelets and rather **cheap/tacky** souvenirs yesterday.
5. The famous spicy **liver/steak** dish requires five **fresh/juicy** tomatoes and three peppers.
6. Sue is a pretty **social/ironic** person but at times her weird **manner/temper** bothers people.
7. She wore an awful **winter/filthy** coat but a nice **golden/trendy** necklace at the party.
8. That girl with **brown/hazel** eyes loved to hear **magic/fairy** tales before going to sleep.
9. They saw the famous **guest/baron** visiting the old and ancient **square/shrine** with some guards.
10. The pretty lady **merely/mildly** petted her cute and really **floppy/fluffy** rabbit after the vets.
11. Jane got a free **tissue/blouse** from a very nice **stand/stall** owner the other day.
12. The brave young **pilot/medic** escaped from a remote **prison/trench** during the war.
13. He replied with some **silly/witty** comments after Jenny **simply/humbly** pointed out the flaw.
14. He set up twice **weekly/yearly** meetings at the modern **round/cubic** building near the sea.
15. In her dreams she met a weird **alien/pixie** while in the street **buying/sewing** nice long skirts.
16. The soldiers were **united/heroic** fighting at the Western **border/bunker** against the enemy.

17. We had a little **break/snack** during the one last **match/derby** between the two rival teams.
18. The senior film **expert/critic** likes to publicise their **latest/cogent** appeals for help.
19. The museum curator **closed/barred** every window to keep the flimsy **paper/satin** models safe.
20. It is a very **clean/dusty** room with a light **green/olive** clock on the wall.
21. I adopted a more **recent/astute** tactic to defend against **bigger/taller** opponents in boxing.
22. Alison is a very **honest/lively** person and has a strong **moral/ethic** about that issue.
23. Due to the recent **crisis/famine** prices will increase by about **double/treble** over the year.
24. He had left a brown **stick/spade** near the path of the leafy **street/suburb** during lunch break.
25. The front of the ornate **stage/villa** covered in dark green **marks/mould** needs fixing up.
26. The old hated **leader/tyrant** really liked taking **taxes/fines** needlessly from his subjects.
27. She saw the nasty **fellow/bandit** carry his sharp and shiny **sword/sabre** towards his victims.
28. They had truly **wanted/seized** power from the angry **fallen/spying** monarch after the coup.
29. He always yells **brief/inane** remarks and shouts **choice/snappy** comments during meetings.
30. He was rather **tired/weary** after all of the stupid **tests/exams** people expected him to sit.
31. He saw the torch **flash/shine** across the bird's hidden **place/perch** while they searched.
32. He would often **repeat/recite** things like some clever **words/prose** written in a novel.
33. It was a fragile **peace/truce** between the two ancient **people/tribes** inhabiting the valley.
34. He had noticed **failed/vulgar** jokers would quite often **blame/amaze** stupid people the most.
35. He washed the dirty **tools/linen** using detergent to remove **black/gravy** stains from last week.
36. His son would slowly **build/carve** models of a famous **dragon/sphinx** every night after school.

37. I heard the moody **author/pastor** pacing around the quiet **avenue/cellar** while he was thinking.
38. He has massive **hidden/unseen** issues which will always **limit/haunt** attempts to make friends.
39. He saw the smart **player/bowler** throw the ball and cause **chaos/havoc** among the opposition.
40. The animal will quickly **attack/pounce** after it slowly creeps **closer/nearer** through the trees.
41. He felt a local **proper/kosher** butcher would always stock **better/richer** meat than other shops.
42. The brave monster **killer/slayer** quickly drove the sharp **point/spear** through his foe's chest.
43. His little sister **easily/neatly** jumps and then swiftly **turns/spins** during her ballet recital.
44. The kind nurse **slowly/calmly** explained that their **future/unborn** children would be fine.
45. There was a woman **asking/raving** about the new fiery **planet/meteor** recently seen in the sky.
46. He held quite **proud/rigid** views about himself and found **valid/snide** criticism very annoying.
47. The robber hid the stolen **money/purse** under the dark brown **sheet/crate** beside the table.
48. Apparently a large **beast/brute** hunts near the river **mouth/brook** during dark nights.
49. The man's cunning **excuse/deceit** allowed him to avoid **paying/parole** after his offence.
50. It was his great **spirit/vanity** which drove the famous **knight/tycoon** through thick and thin.
51. The illness affects **blood/gland** function and makes the patient **worse/vomit** while they sleep.
52. The man cannot **cancel/sprint** because of his awful **health/asthma** issues that plagued him.
53. She bought the lovely **poster/basket** yesterday from the quaint **stores/weaver** near the river.
54. The nice moist **energy/yogurt** bars were made with actual **apple/melon** pieces and oats.
55. We drank lovely **white/amber** drinks and discussed the large **amount/yields** harvested this year.
56. The hidden golden **ticket/coupon** allows free entry to the awesome **theme/koala** park after eleven.

57. The agent was very **direct/abrupt** when discussing the fairly **major/hefty** business deal.
58. The very able **heart/tooth** doctor saw the patients were **living/coping** well with their malady.
59. He knew his pretty **young/needy** wife wanted the room decorated with **yellow/citron** colour paint.
60. Yesterday, the very **prompt/speedy** butler had the sharp **silver/nickel** cutlery laid out.
61. Today, the honest **legal/legit** banker promised he would **shield/redeem** their money for them.
62. The queen looked **royal/regal** when she entered the very **secure/scenic** venue for the banquet.
63. She looked for her office **folder/binder** that had the latest **basic/taxed** order details in it.
64. The really timid **member/tester** admitted that he felt **alive/dizzy** after going on the ride.
65. Warily, the jailed **adult/thugs** agreed to take boring **retail/sewage** jobs when they got out.
66. The poor hapless **family/beggar** near the city **campus/tavern** looked quite lost.
67. She was making a healthy **great/vegan** meal that needed some **taste/onion** added to complete it.
68. In the garden was a brown **chair/canoe** next to the huge **plant/hedge** that needed to be binned.
69. At the annual **party/feast** there was always someone **acting/posing** like a fool.
70. It was rather **common/quaint** that they went to the country **studio/shores** whenever they could.
71. Tom looked **stable/feeble** enough so the consultant cured his poor **motion/joints** with surgery.
72. The cakes were **filled/cooler** with chocolate so they **worked/tasted** great at the party later.
73. He studied the water **output/deluge** seen coming from local **spring/floods** over the last week.
74. He failed with his chosen **career/melody** with hopes of success **ending/fading** with each effort.
75. The writer's words were oddly **stated/poetic** when they were read **today/aloud** with his wife.
76. It was a nice **county/parish** where the councillors were **adding/zoning** more key developments.

77. The gaudy advert **agency**/**flyers** promised that the strange **bottle**/**liquor** would sell well.
78. Pictures that depict **visual**/**morbid** scenes of the past were **forced**/**etched** into the treasure chest.
79. He was asked to closely **survey**/**assess** land around where that **damage**/**fiasco** happened yesterday.
80. It was the calm **master**/**sheikh** that was asked where his rare **degree**/**blazer** came from exactly.
81. He took the shady **route**/**alley** since it led to the blue **river**/**ferry** where he met the others.
82. With their trusty **camera**/**bowman** they had tracked the rare **gopher**/**cougar** around its habitat.
83. He hacked into the secure **domain**/**portal** where he could quickly **delete**/**enrich** photos online.
84. The firm, fussy **female**/**patron** created a system to easily **manage**/**govern** rosters of work shifts.
85. He came up with the precise **scheme**/**sketch** including the very fancy **suite**/**flats** next door.
86. Pressing the stiff **button**/**clutch** meant the system could **switch**/**divert** currents of electricity.

Supplementary references

1. Davis, C. J. N-watch: A program for deriving neighborhood size and other psycholinguistic statistics. *Behav. Res. Methods* **37**, 65–70 (2005).

2. Miller, J., Patterson, T. & Ulrich, R. Jackknife-based method for measuring LRP onset latency differences. *Psychophysiology* **35**, 99–115 (1998).

3. *Percept. Psychophys.* **17**, 578–586 (1975).

4. Rayner, K. The perceptual span and peripheral cues in reading. *Cogn. Psychol.* **7**, 65–81 (1975).

5. Underwood, N. R. & McConkie, G. W. Perceptual Span for Letter Distinctions during Reading. *Read. Res. Q.* **20**, 153 (1985).

6. Degno, F. *et al.* Parafoveal previews and lexical frequency in natural reading : Evidence from eye movements and Fixation-related potentials. *J. Exp. Psychol. Gen.* **148**, 453–474 (2019).
