# Supplementary material for: Neural evidence for lexical parafoveal processing
Source: Nat Commun. 2021 Sep 2;12:5234. doi: 10.1038/s41467-021-25571-x (PMC8413448; doi:10.1038/s41467-021-25571-x)
Supplement: Supplementary file 3 — Reporting summary [file 41467_2021_25571_MOESM3_ESM.pdf]

nature portfolio

Corresponding author(s): Dr. Yali Pan

Last updated by author(s): Jul 8, 2021

Reporting Summary

Nature Portfolio wishes to improve the reproducibility of the work that we publish. This form provides structure for consistency and transparency in reporting. For further information on Nature Portfolio policies, see our Editorial Policies and the Editorial Policy Checklist.

Statistics

For all statistical analyses, confirm that the following items are present in the figure legend, table legend, main text, or Methods section.

n/a	Confirmed	
☐	☒	The exact sample size (*n*) for each experimental group/condition, given as a discrete number and unit of measurement
☐	☒	A statement on whether measurements were taken from distinct samples or whether the same sample was measured repeatedly
☐	☒	The statistical test(s) used AND whether they are one- or two-sided<br>*Only common tests should be described solely by name; describe more complex techniques in the Methods section.*
☒	☐	A description of all covariates tested
☒	☐	A description of any assumptions or corrections, such as tests of normality and adjustment for multiple comparisons
☐	☒	A full description of the statistical parameters including central tendency (e.g. means) or other basic estimates (e.g. regression coefficient) AND variation (e.g. standard deviation) or associated estimates of uncertainty (e.g. confidence intervals)
☐	☒	For null hypothesis testing, the test statistic (e.g. *F*, *t*, *r*) with confidence intervals, effect sizes, degrees of freedom and *P* value noted<br>*Give P values as exact values whenever suitable.*
☐	☒	For Bayesian analysis, information on the choice of priors and Markov chain Monte Carlo settings
☐	☒	For hierarchical and complex designs, identification of the appropriate level for tests and full reporting of outcomes
☐	☒	Estimates of effect sizes (e.g. Cohen's *d*, Pearson's *r*), indicating how they were calculated

Our web collection on statistics for biologists contains articles on many of the points above.

Software and code

Policy information about availability of computer code

Data collection	Stimuli presentation was coded by Psychophysics Toolbox -3 (http://psychtoolbox.org/).<br>MEG data were acquired using a 306-sensor TRIUX Elekta Neuromag system (Elekta, Finland).<br>Eye movement data were acquired from eyeLink 1000 Plus (SR Research Ltd, Canada)<br>Structural MRI images were acquired using a 3T Siemens Prisma scanner (Siemens, Germany).
Data analysis	The data analyses were performed in MATLAB R2019b (Mathworks Inc, USA) by using the FieldTrip toolbox (version 20200220, https://www.fieldtriptoolbox.org/) and custom-made scripts.<br>All the t-tests in this study were two-sided pairwise student's t-tests and were conducted in R (https://www.r-project.org/).<br>Experimental paradigm scripts (Psychtoolbox), statistics scripts (R), scripts to generate all figures are available on OSF (https://osf.io/ard6h/).

For manuscripts utilizing custom algorithms or software that are central to the research but not yet described in published literature, software must be made available to editors and reviewers. We strongly encourage code deposition in a community repository (e.g. GitHub). See the Nature Portfolio guidelines for submitting code & software for further information.

Data

Policy information about availability of data

All manuscripts must include a data availability statement. This statement should provide the following information, where applicable:

- Accession codes, unique identifiers, or web links for publicly available datasets
- A description of any restrictions on data availability
- For clinical datasets or third party data, please ensure that the statement adheres to our policy

Source data are provided with this paper. We have deposited the following data in the current study on figshare (https://figshare.com/account/home#/

projects/117885): the raw MEG data, the epoch data after pre-processing, the raw EyeLink files, the Psychotoolbox data, and the head models after the co-registration of anatomical MRI with the MEG data.

Field-specific reporting

Please select the one below that is the best fit for your research. If you are not sure, read the appropriate sections before making your selection.

[x] Life sciences   [ ] Behavioural & social sciences   [ ] Ecological, evolutionary & environmental sciences

For a reference copy of the document with all sections, see nature.com/documents/nr-reporting-summary-flat.pdf

Life sciences study design

All studies must disclose on these points even when the disclosure is negative.

Sample size	According to an early Rapid Invisible Frequency Tagging study from our group (see below), tagging response from 25 subjects were robust enough for statistical test. So in the current study, we aimed for at least 25 subjects who showed strong tagging response. Due to the smaller tagging patch and shorter tagging duration compared with Zhigalov's study, we recruited 43 subjects to obtain stable tagging response from 26 subjects.<br>Zhigalov, Alexander, et al. "Probing cortical excitability using rapid frequency tagging." Neuroimage 195 (2019): 59-66.
Data exclusions	Four out of forty-three subjects were excluded from analysis due to poor eye tracking accuracy (2 subjects) or falling asleep during the recordings (2 subjects), which left thirty-nine participants.
Replication	We replicated the findings using two different measurements, namely 'the first fixation duration' (Fig2a) and 'the gaze duration' (Supplementary Figure. 1). We also replicated the main neural result in Fig.4 with a subset of the sentence-set in the response to the 1st question from Reviewer 2.
Randomization	In the current study, the main factor was lexical frequency for the target words, which was a within subject design and no participant allocation according to this factor.<br>We only allocated participants for one of the two versions of sentences, and this was done randomly according to the participants code.
Blinding	Investigators were not blind to the sentence version that the participant would read in the experiment (i.e., whether a given sentence frame is embedded with targets of high or low word frequency). Because two sentence versions are just use to counter-balance the frames in different target conditions. Participants were randomly chosen to different sentence versions.

Reporting for specific materials, systems and methods

We require information from authors about some types of materials, experimental systems and methods used in many studies. Here, indicate whether each material, system or method listed is relevant to your study. If you are not sure if a list item applies to your research, read the appropriate section before selecting a response.

Materials & experimental systems

n/a	Involved in the study	
[x]	[ ]	Antibodies
[x]	[ ]	Eukaryotic cell lines
[x]	[ ]	Palaeontology and archaeology
[x]	[ ]	Animals and other organisms
[ ]	[x]	Human research participants
[x]	[ ]	Clinical data
[x]	[ ]	Dual use research of concern

Methods

n/a	Involved in the study	
[x]	[ ]	ChIP-seq
[x]	[ ]	Flow cytometry
[ ]	[x]	MRI-based neuroimaging

Human research participants

Policy information about studies involving human research participants

Population characteristics	We recruited forty-three native English speakers (28 females), aged 18-35 years old (22 ± 2.6, mean ± SD), right-handed, with normal or corrected-to-normal vision, and without a neurological history or language disorder diagnosis.
Recruitment	We recruited participants through flayers and the Psychology Research Participants Scheme for the University of Birmingham. All participants who meet the requirements mentioned above would be included without any self-selection bias or other biases.
Ethics oversight	The University of Birmingham Ethics Committee approved the study.

Note that full information on the approval of the study protocol must also be provided in the manuscript.

Magnetic resonance imaging

Experimental design

Design type	Resting state
Design specifications	N/A
Behavioral performance measures	No behavioral performance involved.

Acquisition

Imaging type(s)	Structural MRI (T1 scans)
Field strength	3T
Sequence & imaging parameters	Time: 4:52<br>Voxel dimensions: 1mm isotropic<br>TR: 2000ms<br>TE: 2.01ms<br>TI: 880ms<br>Flip angle: 8deg<br>FOV: 256x256x208mm<br>GRAPPA factor: 2
Area of acquisition	A whole brain scan was used.

Diffusion MRI ☐ Used ☒ Not used

Preprocessing

Preprocessing software	FieldTrip toolbox (version 20200220, https://www.fieldtriptoolbox.org/)
Normalization	Data were processed with a nonlinear transformation in addition to the linear affine registration.
Normalization template	MNI305 template space
Noise and artifact removal	N/A
Volume censoring	N/A

Statistical modeling & inference

Model type and settings	N/A
Effect(s) tested	N/A

Specify type of analysis: ☒ Whole brain ☐ ROI-based ☐ Both

Statistic type for inference (See Eklund et al. 2016)	N/A
Correction	N/A

Models & analysis

n/a	Involved in the study
☒	☐ Functional and/or effective connectivity
☒	☐ Graph analysis
☒	☐ Multivariate modeling or predictive analysis
